# ﻿A new species of *Monstrilla* (Copepoda, Monstrilloida) in coastal waters of northern South China Sea

**DOI:** 10.3897/zookeys.1232.144830

**Published:** 2025-03-18

**Authors:** Zhiqian Zhou, Xiping Lian, Yehui Tan

**Affiliations:** 1 Marine Biodiversity Collections of South China Sea, Key Laboratory of Tropical Marine Bio-resources and Ecology, South China Sea Institute of Oceanology, Chinese Academy of Sciences, Guangzhou, 510301, China South China Sea Institute of Oceanology, Chinese Academy of Sciences Guangzhou China; 2 University of Chinese Academy of Sciences, Beijing, 100049, China University of Chinese Academy of Sciences Beijing China

**Keywords:** copepods, *Monstrilla*, zooplankton, taxonomy, China seas

## Abstract

A new monstrilloid copepod species, *Monstrillapseudograndis***sp. nov.**, from coastal waters in the northern South China Sea is described and illustrated. The diagnostic characters of this new species include the presence of small setae and sensilla on the forehead and four pairs of nipple-like scars on the ventral surface of the cephalothorax. Although the new species closely resembles *Monstrillagrandis* Giesbrecht, 1891 in overall morphology, it can be distinguished by the shorter antennule, the thumb-like process on the fifth leg, and the distinctive shape of the oral papilla. This represents the eighth record of this genus from China seas.

## ﻿Introduction

The order Monstrilloida Sars, 1901 is renowned for its unique and enigmatic copepods that exhibit a fascinating life cycle transitioning from endoparasitic juveniles to free-living, non-feeding adults (Huys and Boxshall 1991; Suárez-Morales 2011, 2018). Monstrilloids are known to parasitize various marine benthic invertebrates, including polychaetes and molluscs, during their preadult stages (Huys et al. 2007; Suárez-Morales 2011; Suárez-Morales et al. 2014). Adults are characterized by their non-feeding, free-swimming nature, and lack of mouthparts; they are typically encountered in plankton samples from coastal and estuarine environments (Sale et al. 1976; Suárez-Morales 2011; Lee et al. 2016).

In recent decades, the records of monstrilloids have increased rapidly, with seven valid genera, about 183 accepted species, recognized within the single family Monstrillidae Dana, 1849 (Grygier and Ohtsuka 2008; Suárez-Morales 2011, 2018, 2019; Suárez-Morales and Mckinnon 2014; Jeon et al. 2018; Walter and Boxshall 2024). Currently, research on the taxonomy and diversity of Monstrilloida continues to expand globally, with studies covering marine regions of Australia (Suárez-Morales and Mckinnon 2014), Korea (Lee and Chang 2016; Lee et al. 2016; Jeon et al. 2018), China (Lian and Tan 2019), the Philippines (Suárez-Morales 2021), Brazil (Da Cruz Lopes da Rosa et al. 2021), Canada (Delaforge et al. 2017), Mexico (Suárez-Morales 2024; Suárez-Morales and Velazquez-Ornelas 2024), and the Mediterranean Sea (Suárez-Morales et al. 2017). In addition to diversity studies, research has focused on the biology and ecology of monstrilloids, particularly their parasitic behavior, host interactions, and effects on host health, especially on mussels (Suárez-Morales et al. 2010, 2014; Carneiro-Schaefer et al. 2017).

*Monstrilla* Dana, 1849–1852, is one of the most diverse genera within Monstrillidae (Suárez-Morales 2011, 2018; Jeon et al. 2024), and the proportion of antennule and the shape of fifth leg are important identification criteria in the morphological identification of females of this genus (Lee and Chang 2016; Suárez-Morales 2024). *Monstrilla* has a global distribution comprising 53 species to date and includes about 29% of all recorded monstrilloids (Walter and Boxshall 2024). Despite its extensive contribution to marine biodiversity, taxonomic studies on monstrilloid copepods in China are limited (Suárez-Morales 2018), with only seven species of *Monstrilla* having been recorded in the seas of China (Chen and Li 2008; Chen and Huang 2012; Walter and Boxshall 2024). The absence of data on Chinese species may lead to an underappreciation of the diversity of monstrilloids and hinder adequate risk assessments for mussels and other benthic marine invertebrate aquaculture industries.

During a recent re-examination of zooplankton deposited in the South China Sea marine biodiversity collections of the Chinese Academy of Sciences (SCSMBC), an adult female specimen of *Monstrilla* was discovered. This specimen, after taxonomic analysis and comparison with known congeners, was identified as a new species, *M.pseudograndis* sp. nov. This discovery not only contributes to the growing knowledge of monstrilloid diversity but also expands our understanding of the distribution of the genus *Monstrilla*.

## ﻿Material and methods

**Figure 1.**
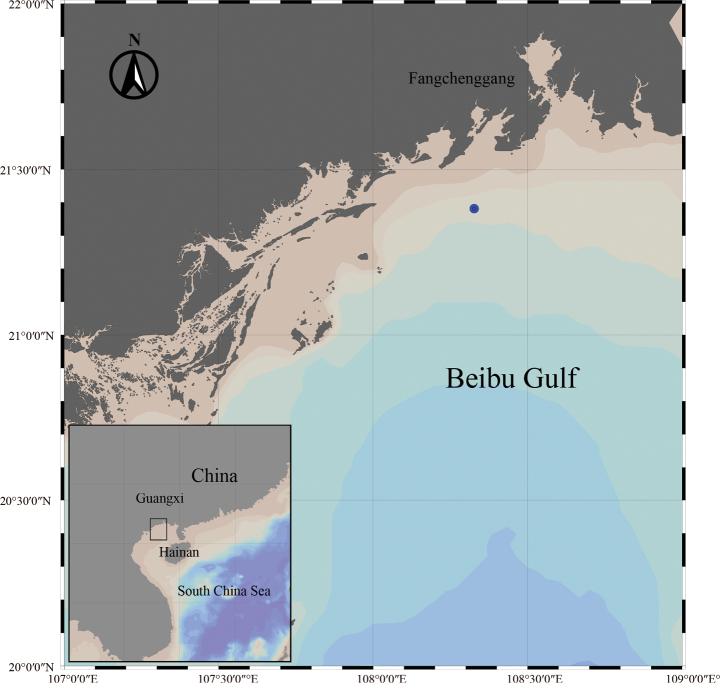
Sampling site of *Monstrillapseudograndis* sp. nov. near Fangchenggang, Guangxi Province, China.

Zooplankton was collected in the coast near Fangchenggang (21°22'53"N, 108°19'38"E), Guangxi Province, China on 7 June 2023 by vertical tow net (0.505 mm mesh, 0.8 m diameter at towing speed of 0.5 m/s) from the surface to a depth of 15 m (Fig. 1). The material was immediately preserved in 5% formaldehyde. Observation and measurements were carried out under a microscope (SMZ18, Nikon, Japan), and drawings were made with the aid of a camera lucida (Leica MC 190HD). Morphologic terminology follows Huys and Boxshall (1991). The nomenclature for the female monstrilloid antennulary armature proposed by Grygier and Ohtsuka (1995) is adopted. The type specimen is deposited in the South China Sea marine biodiversity collections, Chinese Academy of Sciences (**SCSMBC**).

## ﻿Taxonomy

**Subclass Copepoda Milne Edwards, 1840**

**Order Monstrilloida Sars, 1901**

**Family Monstrillidae Dana, 1849**

**Genus *Monstrilla* Dana, 1849–1852**

### ﻿ Monstrilla pseudograndis sp. nov.

Taxon classificationAnimaliaMonstrilloidaMonstrillidae

9851EEA4-949C-5DAF-9365-08704BAE1098

https://zoobank.org/D8D11F64-1FCB-482D-BE80-7DF0CF40076B

**Figure 2.**
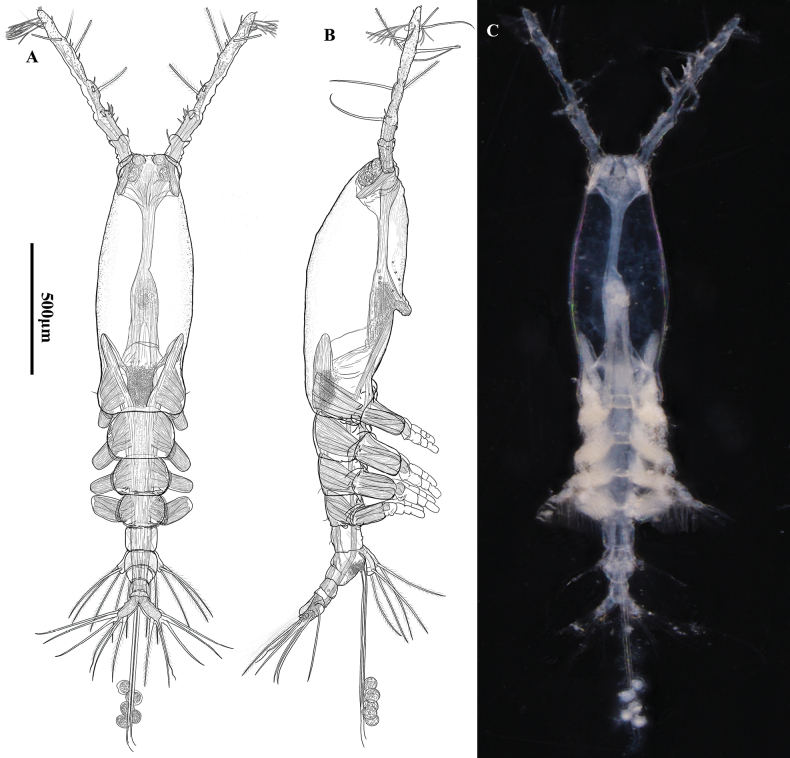
*Monstrillapseudograndis* sp. nov., female holotype **A** habitus, dorsal **B** habitus, lateral **C** habitus, ventral. **A–C** share the same scale bar.

**Figure 3.**
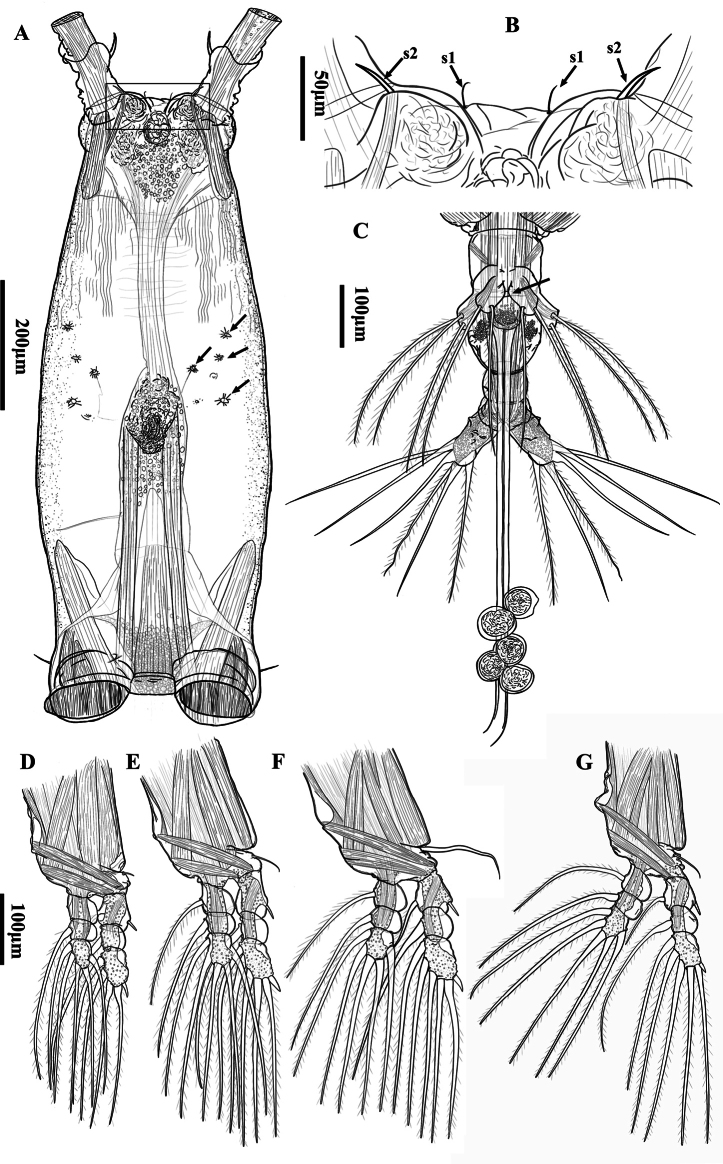
*Monstrillapseudograndis* sp. nov., female holotype **A** cephalosome view, ventral (arrow indicates nipple-like processes) **B** anterior part of cephalothorax, ventral (s1 = sensilla; s2 = setae) **C** postgenital urosomites and caudal rami, ventral **D–G** leg 1–4. **D–G** share the same scale bar.

**Figure 4.**
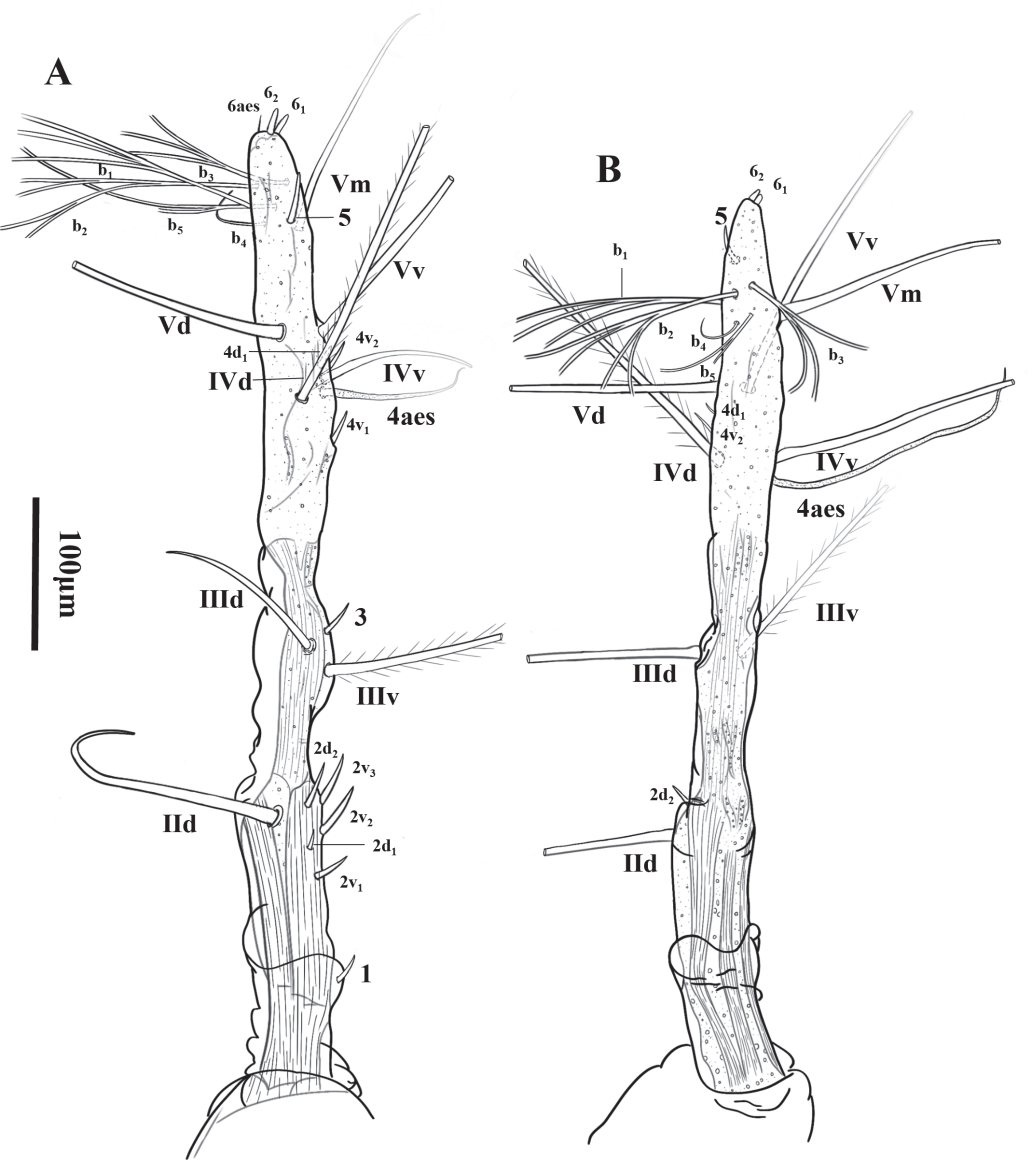
*Monstrillapseudograndis* sp. nov., female holotype **A** left antennule, in dorsal view but tilted little from the medial side **B** right antennule, lateral. **A, B** share the same scale bar.

Figs 2 , 3 , 4 , Tables 1 , 2

#### Type material.

***Holotype***: • adult female (SCSMBC 240185); Zhiqian Zhou leg.; 7 June 2023; partially dissected, formaldehyde preserved.

#### Type locality.

China • Guangxi Province; coast near Fangchenggang. 21°22'53"N, 108°19'38"E; salinity 29.42, temperature 31 °C; depth 17 m.

#### Etymology.

The species name is derived from the Greek word *pseudo*, meaning “false”, and the name of the closely similar *M.grandis* Giesbrecht, 1891.

#### Diagnosis.

Female *Monstrilla* with smooth cuticle on cephalothorax; forehead medially concave, bearing a pair of short sensilla and small setae bilaterally near antennule bases. Cephalothorax ventrally marked by four pairs of small, nipple-like scars, arranged symmetrically anterior to oral papilla. Oral papilla located at approximately midlength of cephalothorax, ventrally posteriorly-bent. Antennule two-segmented, segments fused distally, reaching 37.8% of total body length. Legs 1–4 with relatively short outer exopodal spines. Fifth legs bilobed, outer lobe elongate, with three plumose setae; inner lobe shorter, bearing two plumose setae and a basal protuberance on inner margin. Caudal rami 2.1 times as long as wide, divergent posteriorly, each armed with six well-developed caudal setae.

#### Description of adult female holotype.

Body moderately elongate (Fig. 2A–C), about 1.68 mm, measured from anterior end of cephalothorax to posterior margin of caudal rami, excluding antennules and caudal setae. Cephalothorax rather large and relatively long, accounting for about 57.7% of total body length, transparent, dorsal surface smooth; anterior 2/5 slightly swollen laterally and ventrally. Nauplius eye present, weakly developed, elliptical, ocelli unpigmented with separate oval hyaline bodies, separated by 1½ eye diameter (Fig. 2A). Anteriormost part of cephalothorax with ventral, rounded convex protuberance with irregular margin in lateral view (Fig. 2B); cuticular ornamentation observed on the surface of cephalothorax in lateral and ventral view. Forehead slightly concave medially between antennulary bases in dorsal view, without rostral protrusion, bearing a pair of short, slender sensilla in the middle and a pair of setae near first antennule (Fig. 3B at arrow); weak, fine, longitudinal and transverse wrinkles running behind antennular bases on each side of lateroventral surfaces, flanked by four pairs of small nipple-like scars ahead of oral papilla (Fig. 3A at arrow), without sensory pore. Oral papilla situated slightly posterior to midlength of cephalothorax, accounting for about 52.9%, protru­ding ventrally, with distal half posteriorly-bent (Fig. 3A).

Antennule long (Fig. 4A, B), about 37.8% of total body length, about 65.5% of the cephalothorax; antennule two-segmented, only first segment distinctly separate, remaining segments fused, with constrictions along antennular body representing places of intersegmental divisions (purported 2–5), length ratio of antennule segments, from basal to distal one: 14.0:86.0 (= 100). In terms of the pattern described by Grygier and Ohtsuka (1995) for female monstrilloid antennulary armature, setae (Roman numerals) and spines (Arabic numerals), short, slender element 1 present on first segment; purported segment two with elements 2d_1_, 2d_2_, 2v_1_, 2v_2_, 2v_3_, IId; purported segment three with elements 3, IIId and IIIv; purported segment four with elements 4v_1–2_, 4d_1_, IVd and IVv as well as 4aes (aesthetasc); purported segment five with elements 5, Vd, Vv and Vm; setae b_1–3_, b_5_ all dichotomously branched from proximal half or third, setae b_4_ simple, without b_6_. Apical elements 6_1_, 6_2_, and 6aes present, but 6aes absent on right antennule in dorsal view.

Legs 1–4 (Fig. 3D–G) all with both endopod and exopod three-segmented. Coxa without setae and lacking marginal rows of setae or spines. Basis not fully divided medially from coxa; all outer basal setae on legs 1–4 slender, naked; seta on leg 3 much longer. First and second exopodal segments of legs 1–4 slightly swollen, third exopodal segement undulate along outer distal margin; outer margins of all endopodal segments swollen and smooth. Outer distal spines on first and third exopodal segments of legs 1–4 feeble, much shorter than segments bearing them. Seta/spine armature of swimming legs 1–4 as in Table 1.

**Table 1.** Armature of legs 1–4 including basis, exopods, and endopods in *Monstrillapseudograndis* sp. nov. Roman numerals indicate numbers of spines, and Arabic numerals indicate numbers of setae.

	Coxa	Basis	Endopod	Exopod
Leg 1	0-0	1-0	0-0; 0-1; 1,2,2	I-1;0-1;I,1,3
Leg 2–4	0-0	1-0	0-1; 0-1; 1,2,2	I-1;0-1;I,2,3

Leg 5 bilobed, both lobes confluent basally and divided distally. Outer lobe elongate, armed with three long, plumose setae apically or subapically, of subequal lengths. Inner lobe relatively short, with a basal protuberance on inner margin (arrowed in Fig. 3C), its tip exceeding the half of the outer lobe, armed with two plumose setae apically or subapically.

Urosome consisting of four urosomites: fifth pedigerous somite, genital double-somite, free postgenital somite and anal somite, accounting for 17.9% of total body length, excluding caudal setae; ratio of lengths 30.3:44.0:15.4:10.3 (=100). Genital double-somite representing almost half-length of urosome (44.0%), somewhat swollen laterally, partial suture visible; about 1.7 times longer than the combined length of the next two segments. Genital double-somite bearing pair of long ovigerous spines, these being inserted on middle of ventral surface, basally separated, with pointed tips extending far beyond tips of caudal setae, in total equal to about 38.2% of total body length. Anal somite trapezoidal; lateral margin nearly smooth in dorsal but with apparent notch in ventral; lacking wrinkles or striae both on dorsal and ventral surfaces.

Caudal rami long (Fig. 3C), about 2.1 times as long as wide; divergent outward; with small cuticular protuberance at basal part of outer face and slightly swollen at distal part of inner face; each ramus armed with six well-developed caudal setae, consisting of two distal, two lateral, one inner distal, and one dorsomedial setae.

#### Remarks.

The new species is assigned to the genus *Monstrilla* based on the presence of one free postgenital somite and anal somite in the female, six caudal setae, and the oral papilla located ventrally at nearly midlength of the cephalothorax (Isaac 1975; Huys and Boxshall 1991; Suárez-Morales 2011). Among the females of *Monstrilla*, there are two main types of fifth leg, one of which is formed by a single lobe, such as *M.mariaeugeniae* Suarez-Morales & Islas-Landeros, 1993 (Suárez-Morales and Islas-Landeros 1993). The type II fifth leg is bilobed. Six species, *M.annulata* Suárez-Morales, 2024, *M.cymbula* A. Scott, 1909, *M.gibbosa* Suárez-Morales & Palomares-García, 1995, *M.grandis*, *M.grygieri* Suárez-Morales, 2000, and *M.investigatoris* Sewell, 1949, are similar to the new species in having the type II fifth leg and the antennule that exceeds ½ the length of the cephalothorax (Table 2) (Giesbrecht 1891; Scott 1909; Sewell 1949; Suárez-Morales and Palomares-García 1995; Suárez-Morales 2000b, 2000a, 2024; Chang 2014). Notably, only two species, *M.cymbula* and *M.grandis*, share similar features with *M.pseudograndis* sp. nov., including six well-developed caudal setae on the caudal rami and five setae on the bilobed fifth leg. These three species exhibit the same setation pattern in the fifth leg, a plesiomorphic character state with three exopodal and two endopodal setae, largest number of setae found on the fifth leg in monstrilloids (Huys and Boxshall 1991; Suárez-Morales 2024).

**Table 2.** Comparison of main characters of females in seven similar *Monstrilla* species (elongate cephalothorax and fifth leg with bilobed). Sequence from A to G: *Monstrilla*, *Monstrilla*, *Monstrilla* sp. nov.

Item	*M.annulata*	*M.cymbula*	*M.gibbosa*	*M.grandis*	*M.grygieri*	*M.investigatoris*	*M.pseudograndis* sp. nov.
Antennule length >½ cephalothorax	Yes	Yes	—	Yes	Yes	Yes	Yes
Number of segments of antennule	4	2	3	3	2	2	2
Nauplius eye with pigmented	No	—	No	No	No	Yes	No
Nauplius eye elliptical	—	—	Yes	No	Yes	—	Yes
Oral papilla with posteriorly-bent distal half	Yes	Yes	Yes	No	Yes	—	Yes
Oral papilla near midlength of cephalothorax	No	Yes	No	Yes	Yes	—	Yes
Sensory pores on ventral cephalothorax	No	No	No	Yes	No	No	No
Fifth leg bilobed	Yes	Yes	Yes	Yes	Yes	Yes	Yes
Basal protuberance on inner margin of inner lobe	No	No	No	No	No	No	Yes
Distal thumb-like process on inner margin of outer lobe	No	No	No	Yes	No	No	No
Number of setae on fifth leg	2	5	4	5	4	3	5
Number of setae on caudal rami	5	6	5	6	6	6	6

The forehead between the antennular bases exhibits different cuticular dorsal ornamentations in various species. This area may appear concave with a pair of small setae (Lee and Chang 2016), they may form a protrusion (Suárez-Morales et al. 2013, 2017; Suárez-Morales and Castellanos-Osorio 2019; Suárez-Morales 2024), or they present a flat surface lacking both sensilla and setae (Suárez-Morales and Gasca 2003; Suárez-Morales et al. 2020; Suárez-Morales 2021). Notably, setae and sensilla are either present singly or absent in other species. The new species possesses two distinctive features that render it readily distinguishable among its congeners and support its status as a new member of *Monstrilla*. First, *M.pseudograndis* sp. nov. bears a pair of bilateral setae on the forehead and a pair of medial sensilla (Fig. 3B), marking a unique combination that has not been previously reported in *Monstrilla*. Second, the new species has four pairs of small, nipple-like scars on the cephalothorax, flanked in ventral view, which are commonly recorded as 1–3 pairs in *Monstrilla* (Suárez-Morales 2011, 2018; Jeon et al. 2024).

Among all known species of *Monstrilla*, the new species is most closely related to *M.grandis*, which was reported by Giesbrecht (1891) in the southeastern Atlantic Ocean from southern Patagonia. As a widespread species, *M.grandis* has been extensively redescribed by researchers worldwide (Huys and Boxshall 1991; Suárez-Morales 2000b; Chang 2014). *Monstrillagrandis* and the new species share similar body proportions, including total body length, the position of the oral papilla, and the relative lengths of the cephalothorax and ovigerous spines. In addition to the two distinctive features above mentioned, several significant differences also exist: 1) the relative length of the antennule and the number of segments, which is 37.8% and two in *M.pseudograndis* sp. nov., compared to 48% of the total body length and three in *M.grandis*; 2) the ventral sensory pores of cephalothorax are absent in *M.pseudograndis* sp. nov., whereas they are present in *M.grandis*; 3) *M.pseudograndis* sp. nov. bears a protruding ventral oral papilla, with the distal half posteriorly-bent, in contrast to the slightly protruding, unbent midventral papilla in *M.grandis*.; 4) the setae on the first segment of endopod of leg 2 present in *M.pseudograndis* sp. nov., whereas absent in *M.grandis*; 5) a thumb-like process is absent on the distal part of the inner margin of the outer lobe in *M.pseudograndis* sp. nov., and it is present in *M.grandis*; 6) the inner margin of the inner lobe bears a basal protuberance in *M.pseudograndis* sp. nov., which is absent in *M.grandis*.

## Supplementary Material

XML Treatment for Monstrilla pseudograndis

## ﻿Additional information

### Author ORCIDs

Zhiqian Zhou https://orcid.org/0009-0006-8587-586X

Xiping Lian https://orcid.org/0009-0002-3301-1999

Yehui Tan https://orcid.org/0000-0002-6378-2156

### Data availability

All of the data that support the findings of this study are available in the main text.
